# Single-agent MOR208 salvage and maintenance therapy in a patient with refractory/relapsing diffuse large B-cell lymphoma: a case report

**DOI:** 10.1186/s13256-016-0875-x

**Published:** 2016-05-14

**Authors:** Wojciech Jurczak, Agata Hanna Bryk, Patrycja Mensah, Krystyna Gałązka, Małgorzata Trofimiuk–Müldner, Łukasz Wyrobek, Anna Sawiec, Aleksander B. Skotnicki

**Affiliations:** 1 Department of Hematology, Jagiellonian University, Kopernika 17, Kraków, 31-501 Poland; 2 Department of Pathology, Jagiellonian University, Kraków, Poland; 3 Department of Endocrinology and Nuclear Medicine, Jagiellonian University, Kraków, Poland; 4 Radiological Center, Wyrobek Ltd, Kraków, Poland; 5 Clinical Research Facility – MCM, Kraków, Poland

**Keywords:** DLBCL, CD19, MOR208, MOR00208, XmAb5574

## Abstract

**Background:**

Diffuse large B-cell lymphoma is the most common subtype of non-Hodgkin’s lymphoma. Standard first-line treatment for this aggressive subtype comprises the anti-CD20 antibody rituximab combined with cyclophosphamide, doxorubicin, vincristine, and prednisone. If patients receiving such treatment have an early relapse, or their disease is initially refractory to such treatment, standard salvage regimens may not be effective. There is therefore a high unmet clinical need for new targeted agents that might improve the outcome for such patients. CD19 is a B-lymphocyte lineage-specific cell surface antigen that is expressed by most B-cell non-Hodgkin’s lymphomas. MOR208 is an fragment-crystallizable engineered humanized monoclonal antibody with enhanced antitumor activity that targets CD19 and that may consequently have clinical utility in this setting.

**Case presentation:**

We describe the case of a 33-year-old Caucasian man who presented with a 3-month history of general symptoms and who was admitted to our pulmonology ward with dyspnea due to pneumonia and severe anemia. A histopathological examination of an enlarged right suprasternal lymph node confirmed a diagnosis of T-cell/histiocyte-rich large B-cell lymphoma, an uncommon morphological variant of diffuse large B-cell lymphoma. Our patient had a complete response to first-line rituximab combined with cyclophosphamide, doxorubicin, vincristine, and prednisone, but had an early relapse 5 months after the end of treatment. After intensive salvage therapy consolidated with an autologous stem-cell transplant, our patient again had an early relapse and was subsequently enrolled in a phase IIa trial of single-agent MOR208. Following a scheduled 3 months of weekly treatment, a partial response was confirmed and MOR208 was continued as maintenance therapy, with administration every second week. Positron emission tomography-computed tomography confirmed a complete response 9 months later. This response is ongoing, with a duration of 24 months. MOR208 was well-tolerated by our patient and his quality of life and performance status remain high. No hospitalizations were required and our patient engaged in full-time work and physical activities.

**Conclusion:**

Third-line single-agent therapy with the CD19 antibody MOR208 was highly effective in this patient, despite a history of early relapse after standard first-line and second-line treatment regimens. These data provide support for future randomized studies of MOR208.

## Background

Diffuse large B-cell lymphoma (DLBCL) is the most common subtype of B-cell non-Hodgkin’s lymphoma (NHL), accounting for 30–40 % of cases overall [1]. Immunochemotherapy with the anti-CD20 antibody rituximab, combined with cyclophosphamide, doxorubicin, vincristine, and prednisone (R-CHOP), a current standard first-line treatment for this aggressive subtype, has been shown to increase both response rates and survival compared with CHOP chemotherapy alone [2]. If patients subsequently relapse after such therapy, recommended further treatments in patients of adequate performance status include salvage chemotherapy followed by high-dose chemotherapy and autologous stem cell transplantation (ASCT) [3]. Patients with primary resistance or early relapse within the first 3–6 months after previous therapy have a particularly bad prognosis. Escalating the chemotherapy dose, even high-dose chemotherapy with autologous stem cell support, is less effective in the post-rituximab era than in the pre-rituximab era. In addition, allogeneic transplants may be offered only to selected, fit patients.

Few salvage regimens are effective in patients with DLBCL that relapses soon after, or is refractory to, first-line rituximab plus chemotherapy [4]. There is therefore a clinical need for new targeted agents that might improve outcome for such patients. MOR208 (XmAb®5574, MOR00208) is an fragment crystallizable (Fc)-engineered humanized CD19 monoclonal antibody, which in preclinical studies has shown potent *in vitro* and *in vivo* activity in leukemia and lymphoma model systems. The Fc engineering results in enhanced antibody-dependent cell-mediated cytotoxicity and antibody-dependent cellular phagocytosis compared with the non-engineered parental antibody, as well as direct cytotoxic effects (apoptosis) on tumor cells [5, 6]. CD19 is a type I transmembrane glycoprotein expressed throughout B-cell development and, consequently, by most B-cell NHLs. CD19 is not expressed by most other normal cell types, making this cell surface protein a potentially highly effective therapeutic anticancer target. Indeed, a phase I dose-escalation study showed MOR208 to be safe and well-tolerated with encouraging single-agent activity in patients with relapsed or refractory chronic lymphocytic leukemia (CLL)/small lymphocytic lymphoma (SLL) [7].

In this report, we present the case of a patient with DLBCL, relapsing within the first 6 months after R-CHOP chemotherapy, with a second early relapse experienced after intensive salvage therapy consolidated with ASCT. Our patient was successfully treated with MOR208 in the MOR208C201 clinical trial (EudraCT number: 2012-002659-41; ClinicalTrials.gov identifier: NCT01685008).

## Case presentation

**Fig. 1:**
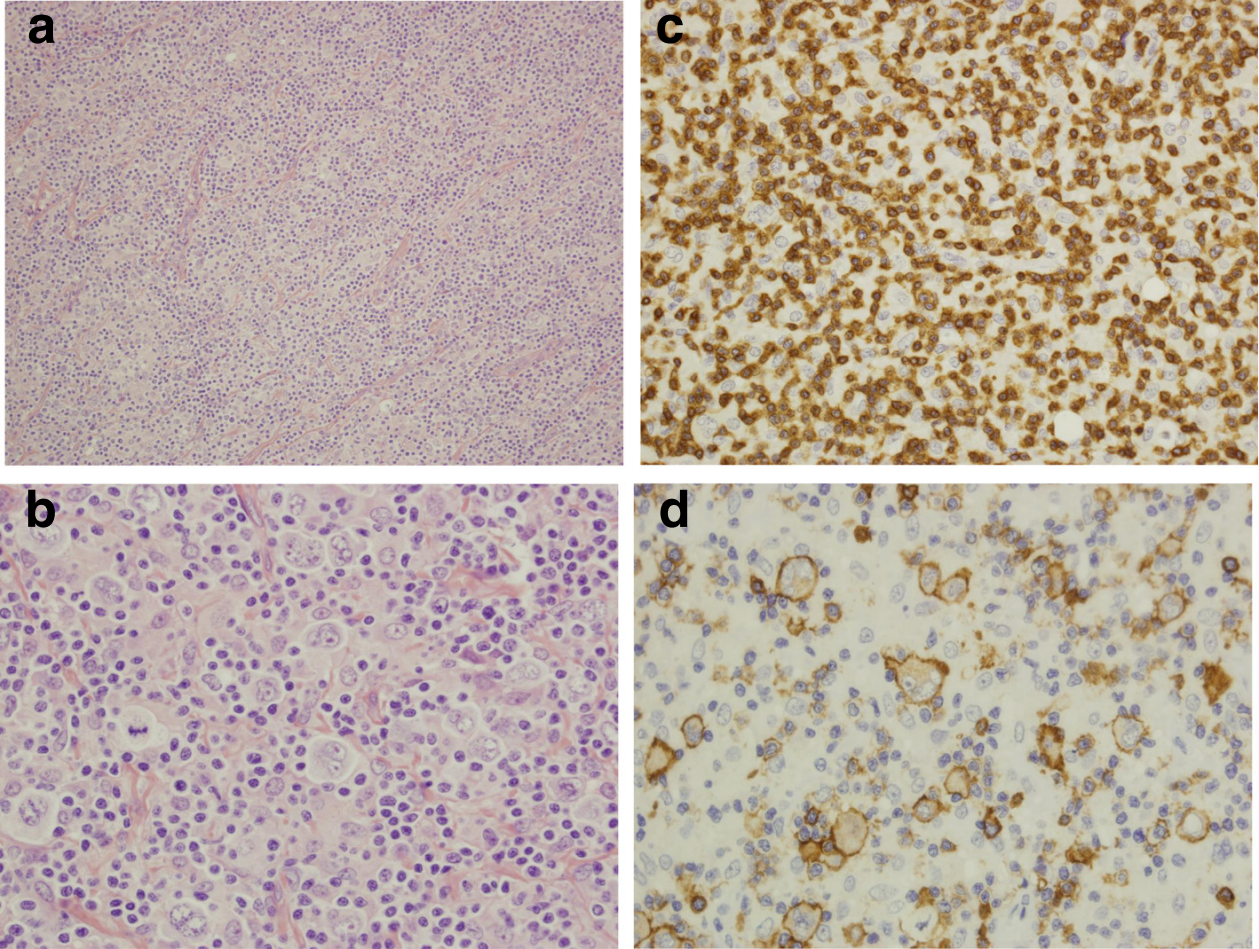
Histopathology. **a** Complete effacement of the lymph node architecture by a diffuse polymorphic cellular population. Atypical large cells dispersed in a background of small lymphocytes and histiocytes. Hematoxylin and eosin (H&E) stain: objective magnification ×20. **b** Dispersed large cells are pleomorphic with irregularly folded nuclei. H&E stain; objective magnification ×60. **c** Small lymphocytes of the background are CD3+ T-cells. Immunohistochemical staining, CD3; objective magnification ×40. **d** Scattered atypical neoplastic lymphocytes with CD20 expression. Immunohistochemical staining, CD20; objective magnification ×60

Our 33-year-old male Caucasian patient presented with a 3-month history of general symptoms (weakness, excessive sweating, and episodes of low-grade fever) and was admitted to our pulmonology ward with dyspnea due to pneumonia and severe anemia (hemoglobin of 8.5 g/dl). A physical examination revealed enlargement of his right suprasternal lymph node, later excised for histopathology (Fig. 1). This confirmed a diagnosis of CD20-positive T-cell/histiocyte-rich large B-cell lymphoma (THRLBCL), an uncommon morphological variant of DLBCL [8]. The neoplastic cells were CD19-positive, partially positive for CD30, and negative for CD15. At diagnosis, routine computed tomography (CT)-based staging confirmed Ann Arbor stage IVB disease, with generalized lymphadenopathy and hepatosplenomegaly (enlarged lymph nodes of the neck [subclavicular on right side], mediastinum [subcarinal, para-aortic, right pulmonary hilum, left pulmonary hilum], right axilla, and abdomen [celiac, para-aortic, para-iliac, spleen]). He also had sclerotic lesions of the ninth, tenth and twelfth thoracic vertebrae. A lumbar puncture with cerebrospinal fluid analysis excluded central nervous system (CNS) lymphoma involvement; in addition, an increased concentration of total protein showed no sign of oligoclonal bands. Our patient was deemed to have an age-adjusted International Prognostic Index (IPI) score [9] of 3 (stage IV, two extra-nodal sites, low lactate dehydrogenase, poor performance status, age <60 years).

**Fig. 2:**
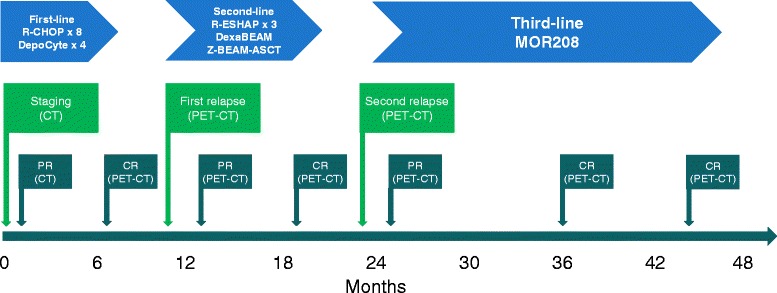
Treatment schema. *CR* complete response; *CT* computed tomography; *DepoCyte* liposomal cytarabine; *DexaBEAM* (dexamethasone, carmustine, etoposide, cytarabine, and melphalan); *PET* positron emission tomography; *PR* partial response; *R-CHOP* rituximab plus cyclophosphamide, doxorubicin, vincristine, and prednisone; *R-ESHAP* rituximab, etoposide, methylprednisolone, cytarabine, cisplatin; *Z-BEAM-ASCT* autologous stem cell transplant conditioned by Z-BEAM (ibritumomab tiuxetan, carmustine, etoposide, cytarabine, and melphalan)

Standard R-CHOP immunochemotherapy was administered to our patient as a first-line regimen (Table 1). As in all high-risk cases, liposomal cytarabine (DepoCyte 50 mg) was administered via the intrathecal route during the first four cycles for prophylaxis of CNS relapse. A good clinical response was confirmed by CT imaging after the third cycle (partial response; PR), followed by a complete response (CR) after eight cycles (treatment schema, Fig. 2) that was confirmed by positron emission tomography-computed tomography (PET-CT). During this first-line therapy, our patient twice developed neutropenic fever, which necessitated prolonged hospital admission, despite granulocyte-colony stimulating factor (G-CSF) primary prophylaxis.

**Table 1:** Treatment regimen for a heavily pretreated patient with diffuse large B-cell lymphoma

First-line treatment	
R-CHOP × 8 intravenously plus liposomal cytarabine (DepoCyte) intrathecally	Rituximab 375 mg/m^2^ (D1) Cyclophosphamide 750 mg/m^2^ (D1) Doxorubicin 50 mg/m^2^ (D1) Vincristine 1.4 mg/m^2^ (D1) Prednisone 40 mg/m^2^/day (D1–D5) Liposomal cytarabine (cycles 1–4)
Second-line treatment	
R-ESHAP × 3	Rituximab 375 mg/m^2^ (D1) Etoposide 40 mg/m^2^/day (D1–D4) Methylprednisolone 500 mg/m^2^/day (D1–D4) Cisplatin 25 mg/m^2^/day (D1–D4) Cytarabine 2000 mg/m^2^ (D5)
DexaBEAM	Dexamethasone 24 mg/day (D1–D10) Carmustine 60 mg/m^2^ (D2) Etoposide 200 mg/m^2^/day (D4–D7) Cytarabine 100 mg/m^2^/day (D4–D7) Melphalan 20 mg/m^2^ (D3)
Z-BEAM-conditioned autologous stem cell transplant	Rituximab 205 mg/m^2^, followed by ibritumomab tiuxetan 32.0 mCi (1184 MBq) (D1) Carmustine 60 mg/m^2^ (D8) Etoposide 200 mg/m^2^/day (D10–D13) Cytarabine 100 mg/m^2^/day (D10–D13) Melphalan 20 mg/m^2^ (D9)
Third-line treatment	
Clinical trial	Induction: MOR208 12 mg/kg for 12 weekly doses Maintenance: MOR208 12 mg/kg every second week

D, treatment day

Early relapse was confirmed 5 months after completion of first-line therapy. PET-CT imaging revealed multiple sites of high standard uptake values (SUVs), including the mediastinum blood pool structure (MBPS) and various sites in his head and neck region (parotid glands, palatine tonsils, submandibular lymph nodes), thorax (supraclavicular lymph nodes on the left, paratracheal chain), and abdomen (para-aortic lymph nodes, stomach, spleen, and liver [Deauville 5]). Elevated SUVs were also detected in his skeletal system, although there was no infection, nor G-CSF used, at that time. Radiological examination also revealed a right-side pleural effusion; in the absence of hypoalbuminemia or heart failure, this was also most likely related to the lymphoma.

As second-line salvage chemotherapy, our patient received three cycles of rituximab, etoposide, methylprednisolone, cisplatin, and cytarabine (R-ESHAP; Table 1). A PET-CT assessment demonstrated a PR to this second-line chemotherapy. A fourth cycle of salvage chemotherapy was then administered: dexamethasone, carmustine, etoposide, cytarabine, and melphalan (DexaBEAM), followed by ibritumomab tiuxetan, carmustine, etoposide, cytarabine, and melphalan (Z-BEAM)-conditioned ASCT (Table 1). This led to a CR, as confirmed by PET-CT. Our patient’s quality of life during second-line treatment and ASCT was poor, due to prolonged hospital admissions and his great concerns relating to potential severe complications of each of the salvage regimens.

At a 3-month follow-up visit after ASCT, our patient presented in good clinical condition and demonstrated weight gain. He had good hematopoietic reconstitution and elevated liver function tests, despite negative tests for hepatitis B and C. Although there were no clinical symptoms of disease or infection, a PET-CT assessment demonstrated increased metabolic activity consistent with early relapse, with high SUVs in the Waldeyer ring (palatine tonsils, pharyngeal tonsil, submandibular lymph nodes), the cervical lymph nodes on his left side, the axillary lymph nodes bilaterally, and his stomach, spleen (with splenomegaly), MBPS, and liver (Deauville 5).

**Fig. 3:**
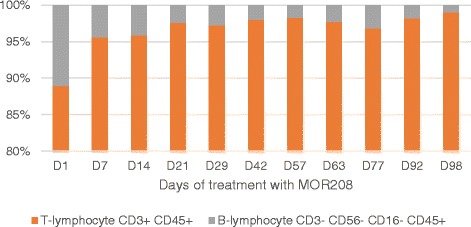
Peripheral blood lymphocyte immunophenotyping by flow cytometry. The percentage of B-lymphocyte and T-lymphocyte counts across treatment time. D, day of treatment with MOR208

**Fig. 4:**
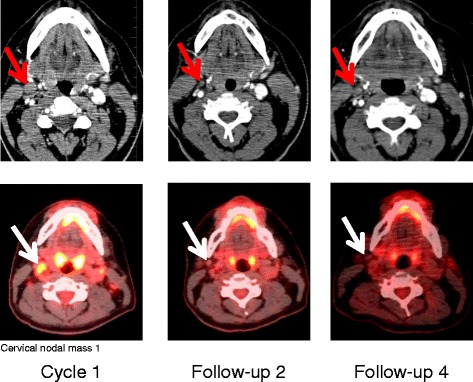
Positron emission tomography-computed tomography scans showing response to single-agent MOR208 therapy (arrow)

At this point, our patient was enrolled in the MOR208 clinical study. During the first 12 weeks of treatment, MOR208 was administered intravenously at 12 mg/kg, once a week. Peripheral blood lymphocyte immunophenotyping by flow cytometry revealed prompt clearance of B-lymphocytes from his peripheral blood; in particular, a decrease in CD3−, CD56−, CD16−, and CD45+ B-lymphocytes and a compensatory increase in CD45+ and CD3+ T-lymphocytes was observed (Fig. 3). Three months after starting third-line MOR208 therapy, PET-CT assessments demonstrated a PR, with only a small number of metabolically active lymph nodes (palatine, cervical on his left side, axillary on his left side, axillary on his right side, lower part of his esophagus, MBPS, liver). Following the scheduled 12 weeks of treatment, MOR208 was continued as maintenance therapy, with administration every second week. PET-CT confirmed a CR 9 months later (Fig. 4).

MOR208 was very well-tolerated, with few adverse events. Our patient developed four upper respiratory tract infections, comprising three instances of pharyngitis that required symptomatic treatment and one incident of bronchitis, which necessitated antibiotic therapy (amoxicillin with clavulanic acid). Our patient did not have any hematological adverse events. During treatment with MOR208, our patient’s quality of life and performance status remained high (World Health Organization grade 0, Karnofsky score 100 %). No hospitalizations were required; all procedures were performed in a day clinic. Our patient engaged in full-time work and physical activities. He continues to be well, with an ongoing duration of response of 24 months.

## Discussion

The original CHOP chemotherapy regimen was introduced by the National Cancer Institute in the mid-seventies. The value of this regimen in the treatment of advanced stage NHL was proven 20 years later in a randomized comparison with so-called second-generation and third-generation regimens [10]. The first and, so far, only indisputable improvement to this regimen has been the incorporation of the anti-CD20 monoclonal antibody rituximab [11]. In particular, the addition of rituximab to CHOP was shown to significantly increase the CR rate, and significantly prolong event-free and overall survival in the first-line treatment of elderly patients with DLBCL. The addition of rituximab to re-induction chemotherapy was also shown to significantly prolong failure-free and progression-free survival in patients with relapsing aggressive CD20+ NHL that had not previously been treated with rituximab [12]. In the pre-rituximab era, high-dose therapy with ASCT allowed the rescue of nearly half of the patients responding to salvage therapy [13]. In the post-rituximab era, the CORAL study in patients with CD20+ relapsed/refractory DLBCL who were randomized to rituximab plus one of two salvage chemotherapy regimens showed that in patients with early relapse (<12 months), prior rituximab treatment was associated with poor prognosis [4]. Patients in this study with the molecular activated B-cell (ABC)-like subtype of DLBCL had a particularly poor outcome, irrespective of the assigned salvage therapy [14].

If NHLs are either initially resistant or subsequently develop resistance to rituximab, targeted agents directed against proteins other than CD20 may improve patient outcome. The B-lymphocyte lineage-specific surface antigen CD19 may represent one such alternative anticancer target. Early-stage studies of a number of CD19-targeted agents have provided encouraging data in patients with CLL and DLBCL [15]. In particular, the CD19 antibody MOR208 provided initial evidence for clinical activity in a phase I dose-escalation study in patients with relapsed or refractory CLL/SLL [7], as well as in the phase IIa NHL study in which our patient is enrolled [16]. In the CLL phase I trial, eight (30 %) of 27 enrolled patients had a PR, and a further 16 had stable disease on the basis of CT criteria, physical examination, and laboratory studies (International Workshop on Chronic Lymphocytic Leukemia criteria [17]). In particular, of the 16 patients treated at the recommended phase II dose of 12 mg/kg weekly, six (38 %) had a PR. The most frequent treatment emergent adverse events (TEAEs) of any grade were infusion-related reactions, which occurred in 11 (67 %) of 27 patients (all grade 1–2), despite premedication. The most common grade 3/4 TEAE was neutropenia (six patients, 22 %). Five patients (19 %) had grade 3/4 treatment-related TEAEs. One drug-limiting toxicity, grade 4 neutropenia lasting ≥7 days, was observed at the maximum administered dose of 12 mg/kg. The objective response rate in the NHL phase IIa trial was 23 % (21 of 92 patients); responses were seen in nine (26 %) of 35 patients with DLBCL, nine (26 %) of 34 patients with follicular lymphoma, and in three (27 %) of 11 patients with other indolent NHLs. Nine (10 %) patients experienced infusion-related reactions that were all grade 1–2 (except for one case of dyspnea, grade 4). Non-hematological and hematological TEAEs of grade 3 or above were seen in 24 (26 %) and 14 (15 %) of 92 patients, respectively. However, our patient did not experience severe hematological or non-hematological TEAEs during treatment with MOR208.

In our patient, we observed an early relapse after first-line R-CHOP chemotherapy, consistent with a dynamic and aggressive lymphoma with a likely poor prognosis. Although our patient underwent salvage chemotherapy followed by ASCT supported by the Z-BEAM conditioning regimen, the disease again proved resistant to treatment, and our patient experienced an early relapse. Considering these rapid relapses at both prior treatment steps, it was deemed to be very unlikely that palliative radiotherapy would have effectively cured the disease and prevented distant metastases. Given the exhaustion of all other available treatment options at our institution, third-line treatment with MOR208 was considered as a possible salvage therapy. Our patient was deemed to be eligible for a phase IIa, open-label, multicenter study of single-agent MOR208 in patients with relapsed or refractory DLBCL. The MOR208 regimen was based on the 12 mg/kg weekly dose recommended in the phase I study [7]. This treatment changed the lymphoma dynamics, possibly preventing its spread to distant sites, and provided extended progression-free survival for our patient (5 months after the R-CHOP chemotherapy and 6 months after BEAM ASCT versus over 20 months during the treatment with MOR208; Fig. 2). His quality of life was excellent and greatly improved compared with previous therapies. He participated in normal daily activities and the treatment was conducted primarily in an outpatient setting.

## Conclusions

The CD19 monoclonal antibody MOR208 was an effective third-line treatment in this patient with DLBCL, despite a history of early relapse following a standard first-line regimen and a second-line treatment regimen that included ASCT. These results support the development of randomized studies of MOR208 therapy in patients with relapsed or refractory DLBCL.

## Consent

Written informed consent was obtained from the patient for publication of this case report and accompanying images. A copy of the written consent is available for review by the Editor-in-Chief of this journal.

## Abbreviations

ABC: activated B-cell
ASCT: autologous stem-cell transplant
BEAM: carmustine, etoposide, cytarabine, and melphalan
CLL: chronic lymphocytic leukemia
CNS: central nervous system
CR: complete response
CT: computed tomography
D: day
DLBCL: diffuse large B-cell lymphoma
G-CSF: granulocyte-colony stimulating factor
H&E: hematoxylin and eosin
IPI: International Prognostic Index
MBPS: mediastinum blood pool structure
NHL: non-Hodgkin’s lymphoma
PET: positron emission tomography
PR: partial response
R-CHOP: rituximab with cyclophosphamide, doxorubicin, vincristine, and prednisone
SLL: small lymphocytic lymphoma
SUV: standard uptake value
TEAE: treatment-emergent adverse event
THRLBCL: T-cell/histiocyte-rich large B-cell lymphoma
